# Laparoscopic Heminephrectomy of Chronically Obstructed Horseshoe Kidney Moiety with Staghorn Calculus, Massive Pyonephrosis, and Xanthogranulomatous Pyelonephritis

**DOI:** 10.1089/cren.2017.0130

**Published:** 2018-03-01

**Authors:** Adrian Fernandez, Benjamin Sherer, Marshall L. Stoller

**Affiliations:** Department of Urology, UCSF Medical Center, San Francisco, California.

**Keywords:** laparoscopy, horseshoe kidney, staghorn calculus, pyonephrosis

## Abstract

Laparoscopic heminephrectomy was performed in a 64-year-old woman with a chronically obstructed horseshoe kidney moiety. More than 3000 cc of pyonephrosis was drained through two percutaneous nephrostomy tubes for infection control before left moiety laparoscopic resection. This case report attests to the feasibility of laparoscopic resection of a massively obstructed horseshoe kidney when performed in a staged manner after prolonged drainage. Since an incision was required for removal of the large specimen, ligation of the horseshoe isthmus was completed through the same incision after hilar control and laparoscopic mobilization of the moiety were completed.

## Introduction and Background

Horseshoe kidneys, resulting from fusion of the metanephric buds at week 4–6 of embryonic development, are the most common renal fusion anomaly, with incidence of ∼0.4 to 1.6 per 10,000 live births.^1^ Compared with the general population, patients with horseshoe kidney have a higher incidence of infection (∼30%) and urolithiasis (∼20%) often because of malrotation and poor drainage or relative obstruction of the ureteropelvic junction.^2,3^ We report an effective management of a chronically obstructed horseshoe moiety with massive pyonephrosis and xanthogranulomatous pyelonephritis.

## Presentation of Case

A 64-year-old Caucasian female with a history of obesity, hypertension, hyperlipidemia, type 2 diabetes mellitus, vitamin D deficiency, and uric acid nephrolithiasis presented with chronic left flank pain, decreased appetite, left abdominal discomfort, and chronic fatigue. On examination, she was found to have a large firm left upper quadrant and flank mass. She was afebrile with stable vital signs.

Initial laboratory evaluation revealed white blood cell (WBC) count of 13.8, Hgb of 9.3, and blood, protein, and WBC esterase positive urinalysis. Urine culture indicated 10,000 CFU/cc of mixed morphotypes of *Escherichia coli*. Creatinine was 1.41 mg/dL.

**FIG. 1.**
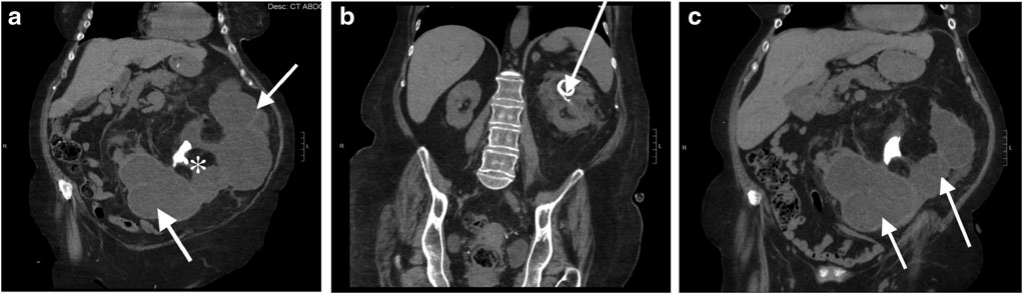
Noncontrast CT images of a 64-year-old woman with chronically obstructed horseshoe kidney. **(a)** Noncontrast CT (May 2016) shows left moiety of horseshoe kidney obstructed with staghorn calculus (*), causing massive pyonephrosis (*arrows*). **(b)** Marked interval improvement in superior pole pyonephrosis of left moiety of horseshoe kidney status postpercutaneous nephrostomy tube placement (*arrow*). **(c)** Persistent severe pyonephrosis (*arrows*) in interpole and inferior pole of left horseshoe kidney moiety prompted placement of second percutaneous nephrostomy tube.

A CT scan of the abdomen and pelvis was obtained, identifying a horseshoe kidney with bilateral nephrolithiasis, including a left-sided staghorn calculus causing obstruction of the ureteropelvic junction and calices. The left moiety had massive caliceal dilation of intermediate density fluid (concerning for pyonephrosis) and thinned parenchyma with perinephric stranding, concerning for xanthogranulomatous pyelonephritis (Fig. 1a). Antibiotics were initiated. A Mag 3 renal scintigram indicated minimal differential function of the left moiety (<2%).

Small stones in the right moiety had attenuation consistent with uric acid, so she was initiated on potassium citrate for urinary alkalinization and protection of the right moiety.

The severe left pyonephrosis was drained through percutaneous nephrostomy tubes, with initial tube placement in the upper pole with >3000 cc drained. Cultures revealed >100,000 *E. coli*, and the patient was continued on culture-specific antibiotics. Repeat CT scan 2 weeks later revealed significant decompression of the left moiety upper pole (Fig. 1b), but an undrained area of the lower pole remained (Fig. 1c), which was managed with an additional percutaneous drain.

Drain outputs, which were significant (>500 cc of purulent fluid per day) for the first few weeks, slowly decreased. After 5 months of recovery, the patient's nutritional status and energy level greatly improved, drain output was minimal, and infection was controlled. Laparoscopic heminephrectomy of the left moiety was planned in a staged manner.

**FIG. 2.**
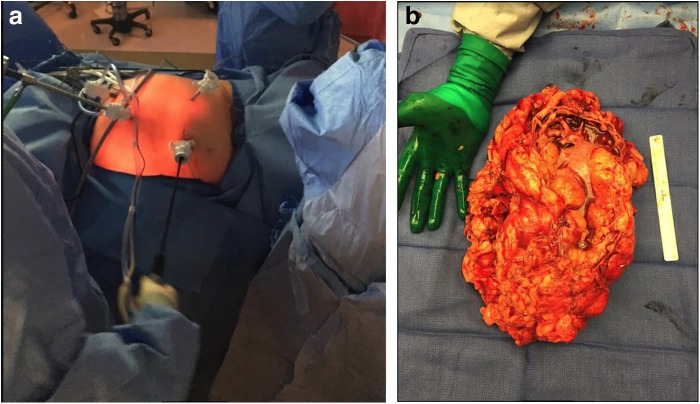
Laparoscopic resection of a massively obstructed horseshoe kidney. **(a)** Four port sites were utilized for laparoscopic dissection of horseshoe kidney. **(b)** Whole, extracted specimen measured 2670 g and 25.5 × 19 × 10.5 cm.

Four port sites were utilized for laparoscopic dissection of the left moiety (Fig. 2a). After lysis of adhesions and complete laparoscopic mobilization of the left moiety and en bloc ligation of the left renal hilum, the only remaining attachment was the isthmus. A low midline incision at the level of the isthmus provided excellent exposure for ligation of the isthmus, which was completed en bloc with a 60 mm Covidien Endo-GIA™ tri-stapler (black load). The massive specimen (Fig. 2b) was extracted through the same low midline incision. Estimated blood loss was 400 mL. There were no intraoperative or postoperative complications. The patient was discharged on postoperative day 3. In postoperative follow-up, the patient endorsed complete symptomatic improvement and renal function from the right moiety was well preserved (postoperative creatinine: 1.03 mg/dL).

## Conclusion

Anatomical anomalies should be considered in the presentation of recurrent nephrolithiasis. Laparoscopic heminephrectomy of a massively obstructed horseshoe kidney is feasible and safe when performed in a staged manner after prolonged drainage and infection control. As an incision is ultimately required for removal of the large specimen, ligation of the horseshoe isthmus can be completed through the same incision after hilar control and laparoscopic mobilization of the moiety are complete.

## Abbreviations Used

CT: computed tomography
WBC: white blood cell
